# Drug-Coated Balloon for De Novo Coronary Artery Lesions: A Systematic Review and Trial Sequential Meta-analysis of Randomized Controlled Trials

**DOI:** 10.1155/2020/4158363

**Published:** 2020-08-10

**Authors:** Wei Liu, Min Zhang, Guangping Chen, Zongzhuang Li, Fang Wei

**Affiliations:** ^1^Department of Cardiology, Guizhou Provincial People's Hospital, China; ^2^Department of Cardiology, Shougang Shuigang General Hospital, China

## Abstract

**Objective:**

To investigate the efficacy of drug-coated balloon (DCB) treatment for de novo coronary artery lesions in randomized controlled trials (RCTs).

**Background:**

DCB was an effective therapy for patients with in-stent restenosis. However, the efficacy of DCB in patients with de novo coronary artery lesions is still unknown.

**Methods:**

Eligible studies were searched on PubMed, Web of Science, EMBASE, and Cochrane Library Database. Systematic review and meta-analyses of RCTs were performed comparing DCB with non-DCB devices (such as plain old balloon angioplasty (POBA), bare-metal stents (BMS), or drug-eluting stents (DES)) for the treatment of de novo lesions. Trial sequential meta-analysis (TSA) was performed to assess the false positive and false negative errors.

**Results:**

A total of 2,137 patients enrolled in 12 RCTs were analyzed. Overall, no significant difference in target lesion revascularization (TLR) was found, but there were numerically lower rates after DCB treatment at 6 to 12 months follow-up (RR: 0.69; 95% CI: 0.47 to 1.01; *P* = 0.06; TSA-adjusted CI: 0.41 to 1.16). TSA showed that at least 1,000 more randomized patients are needed to conclude the effect on TLR. A subgroup analysis from high bleeding risk patients revealed that DCB treatment was associated with lower rate of TLR (RR: 0.10; 95% CI: 0.01 to 0.78; *P* = 0.03). The systematic review illustrated that the rate of bailout stenting was lower and decreased gradually.

**Conclusions:**

DCB treatment was associated with a trend toward lower TLR when compared with controls. For patients at bleeding risk, DCB treatment was superior to BMS in TLR.

## 1. Introduction

Stent implantation is the recommended strategy for majority of coronary artery lesions intended for percutaneous coronary intervention (PCI) [1]. However, stent implantation has several limitations. Long-term follow-up results up to 16 years showed that stent implantation was associated with higher vessel thrombosis and myocardial infarction when compared with plain old balloon angioplasty (POBA) only [2]. Even with the latest generation stent, the rate of major adverse cardiac events was as high as 6.1%, and accompanied with a 2% annual rate thereafter [3]. The persistence of metal material in the vessel wall has been considered one of the causes of adverse events [4]. Therefore, the exploration of a stentless strategy is persistently on the way.

Drug-coated balloon (DCB) is an alternative stentless strategy, which was a combination of angioplasty along with local drug delivery. In 2006, DCB was first introduced to the treatment of in-stent restenosis (ISR) in clinical because it did not involve implanting additional metal layers [5–7]. Afterwards, many studies demonstrated promising results of DCB in the treatment of ISR [8]. In the latest European myocardial revascularization guideline, DCB angioplasty was a Class I recommendation for the treatment of ISR with Level A evidence [1].

Following the successful treatment of ISR, DCB was investigated for its efficacy and safety in de novo coronary artery lesions, based on the hypothesis that foregoing metallic stent implantation in coronary arteries could improve the clinical events [9–13]. Recently, a patient-level meta-analysis compared DCB with non-DCB devices in both de novo and coronary ISR lesions; DCB was associated with a trend toward lower mortality [14]. However, several studies evaluating the DCB for the treatment of de novo coronary artery disease yielded controversial results. Aside from the mixed results, these studies were also not strong enough to conclude the value of DCB in the use of de novo coronary artery lesions. The purpose of the present systematic review and meta-analysis was to evaluate the efficacy of the use of DCB for de novo lesions in different randomized controlled trials (RCTs).

## 2. Materials and Methods

The present systematic review and meta-analysis was performed following the recommendations of the PRISMA (Preferred Reporting Items for Systematic Reviews and Meta-Analyses) statement [15].

### 2.1. Study Protocol

The present study only included RCTs which analyzed PCI with DCB (without stent implantation) versus implantation of bare-metal stents (BMS) and/or drug-eluting stents (DES) or POBA for de novo coronary artery lesions in different clinical settings; this included patients with acute coronary syndrome, stable angina pectoris, high bleeding risk (HBR),or small vessel disease (SVD). The duration of follow-up was 6 months or more. The sample sizes of the studies were not limited.

Several studies were strictly excluded based on the following criteria: (1) studies assessing the efficacy of DCB for the treatment of ISR, (2) studies that analyzed the intervention of DCB in patients with peripheral artery disease or treatment of dysfunctional hemodialysis arteriovenous fistulas, (3) observational studies, registries, and conference abstracts not published formally, such as the PICCOLETO II trial reported in the 2019 TCTmd Conference, and (4) RCTs that compared combination therapy (DCB and BMS or DES implantation) with other strategies (BMS, DES, or POBA).

### 2.2. Search Strategy

PubMed, Web of Science, EMBASE, and the Cochrane Library Database were all searched for eligible studies with publication dates from 2006 up to March 2020, since the first usage of DCB in clinical study was reported in 2006.The following terms were used to perform the PubMed search: (Sirolimus-coated balloon) or (Paclitaxel-coated Balloon) or (drug-coated Balloon) or (Drug-eluting balloon) or (Drug-eluting balloons) or (Drug-coated balloons) and (coronary) or myocardial infarction) and (randomized) or (randomised) or (randomisation) or (randomization). Additional filters, such as the article type and publications dates, were also used. Moreover, we also performed a manual search by scanning the references of the identified articles to find potentially missing studies.

### 2.3. Selection Process and Data Extraction

All potentially relevant studies were independently screened by two authors (WL and MZ). A lot of ineligible studies were excluded according to their titles and abstracts, while the potentially eligible studies had their full texts reviewed. A consensus between the two screening authors needed to be reached in order to determine eligible studies. Any discrepancies were resolved by discussion. The selection process strictly followed the inclusion and exclusion criteria.

Data extraction was independently implemented by the same two authors (WL and MZ). Relevant information from eligible studies was extracted using a prespecified table which contained the relevant items. The following items were extracted from the included studies: comparators (DCB versus DES/BMS/POBA), type of DCB, sample sizes, designation, indication for PCI (acute myocardial infarction [AMI], HBR, SVD, or de novo lesions), duration of follow-up, baseline characteristics (age, gender, and medical history), relevant clinical outcomes, and angiographic outcomes.

### 2.4. Assessment of Study Quality and Risk of Bias

The quality of included studies, which was assessed independently by two investigators (WL and GP C), was evaluated using the Jadad scale. The Jadad scale consists of three items pertaining to descriptions of randomization (0-2 points), double blinding (0-2 points), and dropouts and withdrawals (0-1 point) for a total of five points, with a higher score indicating better quality. Trials scoring 3 or more were considered to be high quality. The Collaboration's Risk of Bias tool was used to assess the risk of bias in included studies.

### 2.5. Statistical Analysis

This study compared both clinical events (TLR, myocardial infarction, and mortality of all causes) and angiographic results (in-segment late lumen loss (LLL) and percent diameter stenosis) for patients treated with DCB versus non-DCB devices. The present study defined TLR as the primary outcome. Risk ratio (RR) or risk difference with a 95% confidence interval (95% CI) was used as a measure of relative risk for the categorical data, such as TLR, mortality of all causes, and myocardial infarction. Mean difference (MD) with the 95% CI was calculated as the effect size for endpoints with continuous data. Either the fixed (Mantel-Haenszel, Rothman-Boice) model or the random effects (inverse-variance) model was adopted to pool the data from each trial, as deemed appropriate. The *I*^2^ statistic and Cochran's *Q* test were used to test statistical heterogeneity. Relevant statistical heterogeneity was considered as Cochran's *Q* test *P* < 0.05 and *I*^2^ > 50%. The fixed effects model was applied to pool the effect sizes if the heterogeneity criteria were not met. Otherwise, the random effects model was used. All the included trials reported events at 6 to 12 months, while only two trials reported events at a 3-year follow-up. Meta-analyses were performed by using data from 6 to 12 months of follow-up, while the events at the 3-year follow-up were depicted qualitatively.

Subgroup analyses were performed based on the comparators (DCB versus uncoated devices and DCB versus DES) and indications of PCI (SVD, HBR, AMI, and de novo lesions). Sensitive analyses were also performed using a leaving-one-out approach. Trial sequential meta-analysis (TSA) was performed to assess the false positive (type I errors) and false negative errors (type II errors).

All meta-analyses were pooled based on the *Cochrane Handbook for Systematic Reviews of Interventions Version 5.1.0*. All statistical analyses were conducted by using Review Manager software version 5.3 (2014, The Nordic Cochrane Centre, The Cochrane Collaboration, Copenhagen, Denmark), and TSA were conducted using TSA software (version 0.9.5.10 Beta). The *P* values less than 0.05 were considered as significant using the 2-sided test.

## 3. Results

**Figure 1:**
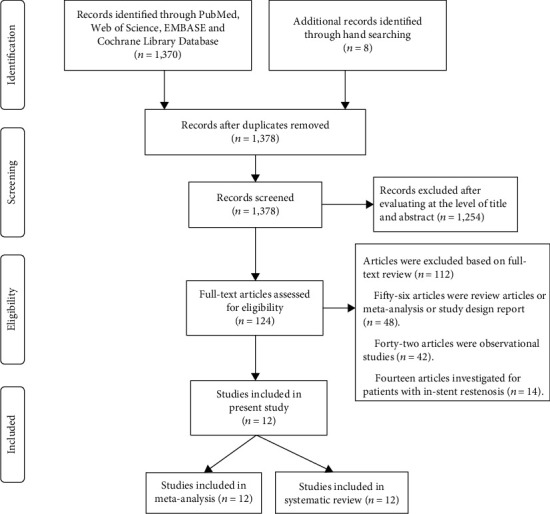
Flow chart of study selection.

Figure 1 illustrates the details of the study search and selection process. Initially, our search yielded 1,378 studies, and 1,254 studies were excluded based on the titles and abstracts. A total of 124 potentially eligible papers further had their full text reviewed. Finally, 12 trials fulfilling the predefined criteria for inclusion were included in thestudy [9–13, 16–22].

In total, 12 RCTs including 2,137 patients were analyzed. All the DCB were paclitaxel coated. Among these, seven trials with 1,482 patients compared DCB and DES, one trial with 210 patients compared DCB and either BMS or DES, and the other four trials compared DCB and uncoated devices (two with POBA and two with BMS). In the present study, we only included patients undergoing PCI with de novo lesions. The clinical presentations of the patients varied. The most common indication was a small vessel lesion seen in five trials, followed by HRB seen in two trials. Majority of trials had 6 to 12 months follow-up, except for the BELLO and DEBUT trials, wherein the duration of follow-ups was 3 years [10, 23]. The baseline characteristics of included trials were summarized in Table 1.

### 3.1. Study Quality and Risk of Bias

**Figure 2:**
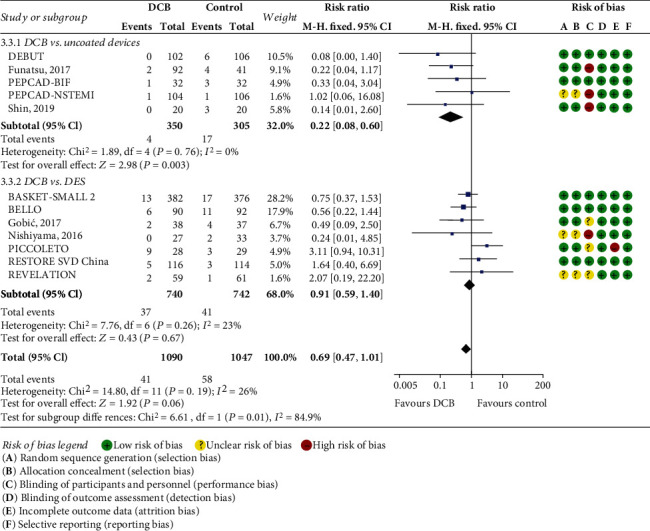
Comparison of the risk of TLR between DCB and controls, and the risk of bias among included studies. Subgroup analysis was performed according to the control group. Uncoated devices included BMS and POBA. BMS: bare-metal stents; POBA: plain old balloon angioplasty; DES: drug-eluting stents; DCB: drug-coated balloons; TLR: target lesion revascularization.

All the included trials were of high quality, with Jadad scores of 3 points or more (Table 1). A summary assessment of the risk of bias is illustrated in Figure 2. The quality was “high” because most information is from RCTs with a low risk of bias.

### 3.2. The Incidence of Target Lesion Revascularization

TLR was evaluated in all the 12 included trials with a total of 2,137 patients. Among these, 1,090 patients were grouped into DCB treatment, and the other 1,047 patients were treated with non-DCB devices. The pooled result showed that there was no significant difference in the incidence of TLR between DCB and non-DCB treatment at 6 to 12 months of follow-up. But DCB treatment was associated with a numerically lower TLR risk (Figure 2. RR: 0.69; 95% CI: 0.47 to 1.01; *P* = 0.06; TSA-adjusted CI: 0.41 to 1.16).

A subgroup analysis of DCB versus uncoated devices (POBA or BMS) revealed that DCB treatment yielded better TLR compared with uncoated devices (Figure 2; RR: 0.22; 95% CI: 0.08 to 0.60; *P* = 0.003). Another subgroup analysis including the DEBUT trial and the study by Shin et al. revealed that DCB treatment was associated with a lower incidence of TLR (RR: 0.10; 95% CI: 0.01 to 0.78; *P* = 0.03) in patients with HBR compared with BMS.

Sensitive analysis after excluding the PICCOLETO study showed that the incidence rate of TLR was significantly lower in DCB treatment compared with non-DCB devices (RR: 0.57; 95% CI: 0.37 to 0.86; *P* = 0.007), which hinted that the PICCOLETO study caused the discrepancy. This was possibly because the PICCOLETO study was prematurely stopped due to high major adverse cardiac event rates in the DCB group. In this study, the inferior results of DCB compared to DES were attributed to the first-generation Dior DCB (Eurocor Tech, Bonn, Germany), with a lower concentration of paclitaxel coated on the balloon [13].

**Figure 3:**
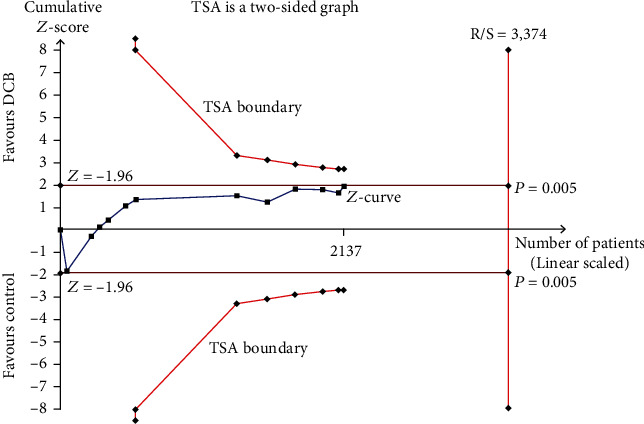
The trial sequential meta-analysis (TSA) on target lesion revascularization. TLR: target lesion revascularization; DCB: drug-coated balloon.

TSA of all trials (type I error 5%; power 80%, relative risk reduction 30%) showed that the required information size was 3,374, which meant that 1,000 more patients need to be randomized before firm conclusions can be drawn regarding the effect on TLR (Figure 3).

### 3.3. The Impact of DCB Treatment on Mortality of All Causes and Myocardial Infarction

At 6 to 12 months of follow-up, no significant differences were observed between DCB and non-DCB devices in mortality of all causes (12 RCTs with 2,137 patients, RD: -0.00; 95% CI: -0.02 to 0.01; *P* = 0.52). A subgroup analysis revealed that DCB treatment was associated with lower mortality of all causes compared to uncoated devices (RD: -0.03; 95% CI: -0.06 to 0.00; *P* = 0.05). Mortality of all causes was similar between the DCB and DES groups. Another subgroup analysis showed that mortality of all causes was concordant in the DCB and non-DCB groups for patients with SVD, HBR, AMI, and de novo lesions. The direction of the results remained unchanged when removing individual studies from the analysis.

DCB treatment was associated with significantly lower incidence of myocardial infarction at 6 to 12 months of follow-up (12 RCTs with 2,137 patients, RR: 0.40; 95% CI: 0.22 to 0.75; *P* = 0.005); for every 50 patients treated, one myocardial infarction is prevented. A subgroup analysis showed that this decreased incidence was mainly driven by its comparison with uncoated devices (RR: 0.13; 95% CI: 0.02 to 0.73; *P* = 0.02). The pooled subgroup analysis showed that DCB treatment had a 61% reduction of myocardial infarction risk in SVD and HBR patients (RR: 0.39; 95% CI: 0.20 to 0.79, *P* = 0.008). The pooled results remained stable after discarding the PICCOLETO study (RR: 0.36; 95% CI: 0.19 to 0.70; *P* = 0.002).

### 3.4. The Qualitative Description of Clinical Results at 3-Year Follow-Up

Only the BELLO and DEBUT studies reported the clinical events at 3-year follow-up, and quantitative analyses were not conducted. The BELLO study enrolled 163 patients with lesions located in the small vessels (reference diameter < 2.8 mm). It found that the use of DCB appears to be associated with lower incidence of major adverse cardiac events (MACE) when compared with DES treatment at 3 years, while no significant differences were observed on TLR. In the DEBUT study, 210 patients with HBR and an ischemic de novo lesion in either the coronary artery or a bypass graft were included. The outcomes showed the proportion of MACE in the DCB group was lower than in the BMS group at 3-year follow-up.

### 3.5. Angiographic Results at Follow-Up

**Figure 4:**
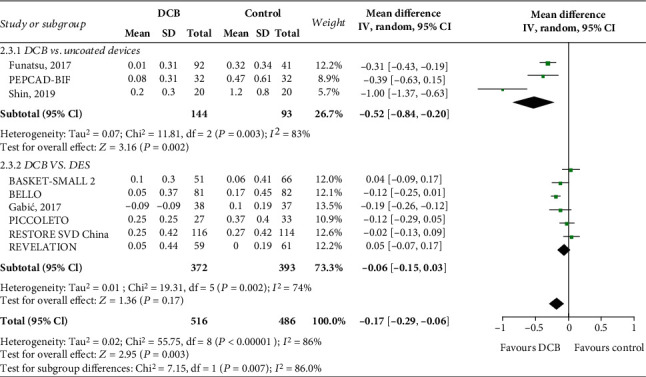
Mean difference in LLL between the DCB and control groups. Subgroup analysis was performed according to the control group. Uncoated devices included BMS and POBA. BMS: bare-metal stents; POBA: plain old balloon angioplasty; DES: drug-eluting stents; DCB: drug-coated balloons; LLL: late lumen loss.

The durations of angiographic follow-up were 6 to 9 months in all the included studies. LLL was reported in nine trials with 1,002 patients. The pooled result showed that DCB treatment was superior to non-DCB devices in terms of LLL with a MD of -0.17 mm with significant heterogeneity (Figure 4; MD: -0.17; 95% CI: -0.29 to -0.06; *P* = 0.003; *I*^2^ = 86%). Subgroup analyses revealed that LLL was significantly lower in DCB treatment compared with uncoated devices (MD: -0.52; 95% CI -0.84 to -0.20; *P* = 0.002), but no difference of LLL was observed between the DCB and DES groups (MD: -0.06; 95% CI -0.15 to 0.03; *P* = 0.17).

Eight trials with 864 patients compared the percent diameter stenosis between the DCB and non-DCB groups. A similar percent diameter stenosis was identified between the DCB and non-DCB groups. Significant heterogeneity was also identified between trials, with *I*^2^ = 87% (MD: -1.55; 95% CI: -8.34 to 5.24; *P* = 0.65; *I*^2^ = 87%). In a subgroup analysis, DCB treatment had a significant benefit when compared with uncoated devices but was inferior to DES.

Sensitive analyses using a leave-one-out approach showed that the overall results of our study for LLL and percent diameter stenosis remained stable.

### 3.6. The Rates of Bailout Stenting in Patients Undergoing DCB Treatment

**Figure 5:**
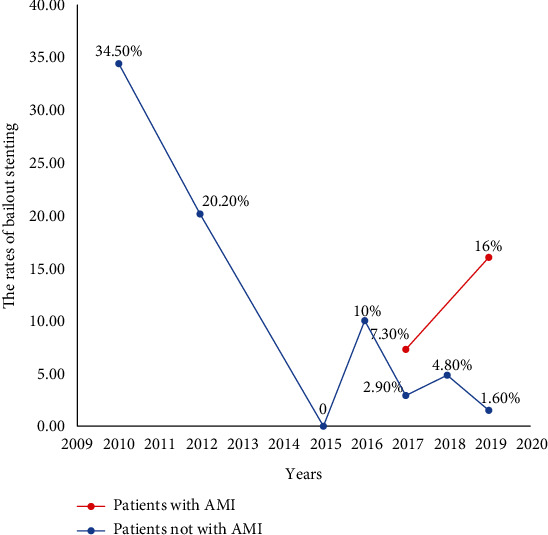
The rates of bailout stenting in patients undergoing DCB treatment. The blue curve showed the trajectory of change in patients not with AMI, and the red one showed the change in patients with AMI. DCB: drug-coated balloons; AMI: acute myocardial infarction.

The rates of bailout stenting varied from 0% to 34.5% among studies (Table 1). Interestingly, we found that the rates of bailout stenting were higher in earlier studies, such as the PICCOLETO and BELLO trials [12, 13], than those in later studies, and gradually decreased (Figure 5). In the recent 3 years, the rate of bailout stenting was less than 5%, and studies in patients with AMI also had higher bailout stenting (Figure 5). These data might display the obvious learning curve of the operation for DCB treatment.

## 4. Discussion

The efficacy and safety of DCB have been demonstrated in the treatment of ISR, and it is recommended as the first-line treatment for ISR in the latest ESC guidelines [1]. Emerging evidence also suggests that DCB may also be useful in de novo coronary artery lesions in patients with SVD and HBR. However, a security crisis of DCB was raised by a recent meta-analysis including 28 RCTs which showed an increased mortality following the application of paclitaxel-coated balloons and stents in the femoropopliteal artery of the lower limbs [24]. Interestingly, in this meta-analysis, the mortality was not high during the first 12 months, but only afterwards. Recently, an individual patient data meta-analysis further confirmed the risk of DCB in lower limbs, with an absolute 4.6% increased mortality risk after 5 years [25].

In contrast to the usage of DCB in lower limbs, the outcomes from our study showed that DCB treatment for de novo coronary lesions did not raise the incidence rates of TLR, mortality of all causes, and myocardial infarction. In fact, our study exhibited a numerical reduction of TLR in patients treated with DCB at 6 to 12 months follow-up, when compared to controls. A subgroup analysis showed that DCB treatment was associated with a lower rate of TLR compared with those treated with uncoated devices (BMS or POBA) and with similar TLR compared to DES treatment. For patients with HBR, the pooled results from DEBUT trial and study by Shin et al. showed that DCB treatment was superior to BMS in terms of TLR rate. Furthermore, the rate of myocardial infarction was also decreased in patients treated with DCB. Angiographic results showed the LLL was significantly reduced in patients treated with DCB. These results reassure the safety of DCB when used in de novo coronary lesions. Since the meta-analysis was inconclusive according to the TSA, we should cautiously interpret its results, and more studies are needed to draw more definite conclusions.

The use of PCI for coronary artery disease (CAD) has had a history of more than 40 years. In 1977, Grüntzig performed the first human percutaneous transluminal coronary balloon angioplasty [26]. The use of POBA, as it is now called, was the first step towards modern coronary interventions. However, the following studies found that the occurrence of the arterial recoiling process, acute closure due to arterial dissection, and renarrowing of the dilated segment after balloon dilatation were apparent in CAD patients treated with POBA [27]. To address the aforementioned problems, BMS and DES were successively introduced to treat patients with coronary stenosis [28]. Currently, the second-generation DES is widely used and has a relatively lower restenosis and MACE compared with POBA, BMS, and first-generation DES [29]. Nevertheless, patients treated with second-generation DES will have the mental and polymer material remain in the vessel wall, both of which could promote chronic inflammation, neoatherosclerosis within the stent, and impaired vasomotor function [4]. Therefore, the concept of leaving nothing behind has been brought up.

The present study showed that DCB was a useful strategy for leaving nothing behind, but aside from this, bioresorbable vascular scaffolds (BVS) are another potential approach to achieve the same goal. BVS provide a temporary scaffolding effect and are then absorbed within a certain period. However, existing evidence indicates that BVS is not applicable for the treatment of CAD so far. The recent ABSORB III trial showed that the adverse event rates, particularly target vessel myocardial infarction (8.6% vs. 5.9%; *P* = 0.03) and device thrombosis (2.3% vs. 0.7%; *P* = 0.01), were higher with BVS than everolimus-eluting stents (EES) at the 3-year follow-up [30]. In accordance with this trial, a recent meta-analysis including 10,510 patients showed that BVS were associated with higher rates of target lesion failure and scaffold thrombosis between 1 and 3 years and cumulatively through 3 years of follow-up compared with EES [31]. Accordingly, the FDA has recently issued an alarm about the use of BVS, citing stent thrombosis as the main concern. The present study, by highlighting the safety of DCB, confers a positive impact and better expectations regarding the stentless strategy.

Several advantages of DCB treatment for de novo coronary lesions have been mentioned. These advantages consist of (1) avoiding the persistence of metal material in the vessel wall, (2) reducing the duration of dual antiplatelet therapy, and (3) allowing for repeatability of the procedure. Because of these advantages, plenty of patients with de novo coronary artery lesions have received DCB treatment. A real-world observational study from the SCAAR registry found that treatment with DCB was associated with significantly lower risk for target lesion thrombosis (adjusted HR: 0.18; 95% CI: 0.04 to 0.82, *P* = 0.03) using a propensity-matched analysis compared to new-generation DES [32]. However, the possible vascular elastic recoil and dissections are still concerns regarding DCB treatment. Notably, our study showed that DCB treatment was associated with a reduced LLL, which meant that vascular elastic recoil and dissections might not be evident. Furthermore, our study reviewing 12 RCTs systematically showed that the rate of bailout stenting was lower, and gradually decreased by the year, with a less than 5% rate of bailout stenting in the past three years for patients without AMI. An interesting phenomenon found in our present study was that the rate of bailout stenting was higher in patients with AMI compared to those without. The possible reasons were as follows: (1) the target local lesion was more vulnerable and unstable in AMI patients, and (2) the PCI procedure was more emergent and urgent, and operators had less time to perform the elaborate operation. Therefore, due to the improvements in operation skills for PCI, DCB treatment, and intravascular imaging technology, the incidence of vascular elastic recoil and dissections which cause bailout stenting was comparatively lower and more acceptable in the current PCI era.

The advancements of DCB technologies facilitated the treatment of DCB for patients with de novo coronary lesions. The sensitive analysis result from our study showed that the PICCOLETO study affected the overall effect significantly. After omitting this study [13], the rate of TLR was lower in the DCB group than in the non-DCB group. The PICCOLETO study used the first-generation Dior DCB (Eurocor Tech, Bonn, Germany), which had a lower concentration of paclitaxel covered on the balloon; this was considered the reason why DCB yielded inferior results compared to DES in this study [13]. Furthermore, several newer generation DCB have shown noninferior or superior results in patients with de novo coronary lesions compared with non-DCB devices [17, 18]. These studies pointed out the fact that not all DCB are equal, and that they cannot be treated as a “class effect.” Future DCB with improvements in excipient technology and introduction of more suitable antiproliferative drugs are expected to improve the treatment of patients with CAD.

Our study found that DCB treatment was superior to BMS in terms of TLR for patients with HBR. Major bleeding was a common complication of dual antiplatelet therapy (DAPT), especially in patients with HBR, and a powerful predictor of morbidity and mortality after PCI [33]. BMS with 1 month DAPT was once recommended [10]. After the emergence of new evidence, new-generation DES with shorter DAPT (3 months for stable CAD and 6 months for acute coronary syndrome) was preferred over BMS for patients with HBR [1]. The LEADERS FREE study had shown that polymer-free DES was superior to BMS with respect to the primary safety and efficacy end points among patients with HBR when used with a 1-month course of DAPT [34]. However, the optimal technique of PCI in patients with HBR is still unknown. The superiority of DCB compared with BMS from our study offered a useful alternative therapy for HBR patients. With short DAPT needed for both DCB and new-generation DES therapy, future studies are warranted to evaluate the efficacy and safety between the two strategies for HBR patients.

## 5. Limitations

Our study has some limitations. First, we only qualitatively reviewed the long-term results of two trials reporting the long-term clinical events [10, 12]. The BELLO study showed the rate of MACE was lower in the DCB group compared with the DES group at 3 years [23]. In the DEBUT study, DCB treatment was associated with lower MACE compared with BMS treatment in patients with HBR at 3-year follow-up [10]. More trials are needed to confirm the long-term efficacy and safety of DCB treatment. Second, bailout stenting was common in the earlier studies, and gradually declined. We could not assess the impact of cross-over treatment systematically since this information was not provided in most of the publications. Third, different types of DCB were used in the available studies. Majority (8 of 12 studies) used SeQuent Please DCB, while Dior, IN.PACT Falcon, Restore, and Pantera Lux DCB were used in one study each. Sensitive analyses performed with a leaving-one-out approach showed that the PICCOLETO study using Dior DCB affected the results, hinting at the potential discrepancies among different DCB technologies. Following the advances of DCB technologies and operators' experience, the efficacy of DCB treatment further improved. Nonetheless, it was inappropriate to conduct an additional analysis comparing different DCB technologies because of the limited data in the present study. Furthermore, the information on concomitant medication, such as antiplatelet and statin therapy, was insufficiently supplied and could therefore not be analyzed. Finally, this is not a patient-level meta-analysis, which may increase the risk of bias; caution must be taken in interpreting the outcomes of the present study.

## 6. Conclusion

DCB treatment had a numerically lower rate of TLR compared to non-DCB devices in patients with de novo coronary artery lesions. TSA showed that more patients were needed to confirm this result. Subgroup analyses showed that DCB was superior to uncoated devices (POBA and BMS) in terms of TLR. No significant differences were observed between the DCB and DES groups. In patients with HBR, DCB treatment had a lower rate of TLR than BMS. More high-quality randomized trials with long-term follow-ups are needed to further evaluate the role of DCB for the treatment of de novo lesions.

## Figures and Tables

**Table 1:** The baseline characteristics of included randomized clinical trials.

Study, year	Sample size, DCB/control	Patients' indication	Types of DCB	Bailout stenting	Control group	Male (%), DCB/control	Age		HBP (%), DCB/control,	DM (%), DCB/control	Maximum FU	Jadad scores
							DCB	Control				
BASKET-SMALL2, 2018 [18]	382/376	SVD	SQP	5.10%	PES/EES	77%/70%	67.2 ± 10.3	68.4 ± 10.3	85%/89%	32%/35%	12 months	3
BELLO, 2012 [12]	90/92	SVD	IN.PACT falcon	20.20%	PES	80%/77.2%	64.8 ± 8.5	66.4 ± 9.0	80%/81.5%	43.3%/38.0%	3 years	3
DEBUT, 2019 [10]	102/106	HRB	SQP	2%	BMS	62%/64%	77.6 ± 8.4	76.2 ± 8.5	87%/91%	26%/49%	3 years	3
Funatsu，2017 [20]	92/41	SVD	SQP	2.90%	POBA	78%/68%	68 ± 10	69 ± 11	84%/73%	48%/32%	6 months	3
Gobić, 2017 [19]	38/37	STEMI	SQP	7.30%	SES	70.7%/73%	57.2 ± 13.1	54.3 ± 10.6	31.7%/35.1%	4.9%/10.8%	6 months	3
Nishiyama, 2016 [21]	27/33	De novo	SQP	10%	EES	74.1%/72.7%	67.3 ± 11.1	70.6 ± 8.97	76.7%/90%	40.0%/43.3%	8 month s	3
PEPCAD-BIF, 2015 [22]	32/32	BIF-SVD	SQP	0%	POBA	75.0%/71.9%	66 ± 12	69 ± 10	NA	34.4%/37.5%	9 months	3
PEPCAD-NSTEMI, 2019 [9]	104/106	NSTEMI	SQP	14.60%	BMS/DES	66.3%/67.9%	66.0 ± 11.4	67.0 ± 13.1	78.7%/87.7%	26.9%/35.8%	9 months	3
PICCOLETO, 2010 [13]	28/29	SVD	Dior	34.50%	PES	78.6%/75.9%	68 ± 9	67 ± 10	75.0%/70.8%	37.9%/46.4%	9 months	3
RESTORE SVD China, 2018 [17]	116/114	SVD	Restore	4.00%	ZES	66.4%/77.2%	60.1 ± 10.5	60.5 ± 10.8	67.2%/75.4%	39.7%/42.1%	1 year	3
REVELATION, 2019 [11]	59/61	STEMI	Pantera Lux	18%	SES	87%/87%	57.4 ± 9.2	57.3 ± 8.3	30%/32%	13%/17%	9 months	3
Shin, 2019 [16]	20/20	HRB	SQP	0%	BMS	70.0%/75%	57.5 ± 9.2	61.6 ± 9.5	40%/45%	35%/25%	12 months	3

Drug-coated balloons investigated: SQP=SeQuent Please (B.Braun, Melsungen, Germany), In.Pact Falcon (Medtronic, Galway, Ireland), Pantera Lux (Biotronik, Berlin, Germany), Dior (Eurocor, Bonn, Germany), and Restore (Cardionovum, Bonn, Germany). BMS: bare-metal stent; DES: drug-eluting stent; EES: verolimus-eluting stent; FU: follow-up; HBR: patients at high bleeding risk; NSTEMI: non-ST-segment elevation myocardial infarction; PES: paclitaxel-eluting stent; POBA: plain old balloon angioplasty; BIF-SVD: side branch bifurcation lesion with small vessel disease; SES: sirolimus-eluting stents; STEMI: ST-segment elevation myocardial infarction; SVD: small vessel disease; ZES: zotarolimus-eluting stent; HBP: hypertension; DM: diabetes mellitus; NA: not available.

## Data Availability

The data used to support the findings of this study are available from the corresponding author upon request.
